# Expert consensus on the terminology, diagnostics and management of persisting symptoms after concussion with a focus on mental health, postural stability, electroencephalogram and balance testing: A cross-sectional Delphi-like survey

**DOI:** 10.17159/2078-516X/2024/v36i1a17870

**Published:** 2024-07-15

**Authors:** MJ Lumb, N Snegireva, K Welman

**Affiliations:** Division of Movement Science and Exercise Therapy, The Movement Laboratory, Department of Exercise, Sport & Lifestyle Medicine, Faculty of Medicine and Health Sciences, University of Stellenbosch, Stellenbosch, Western Cape, South Africa

**Keywords:** brain, contact sport, diagnostics, head injuries, mental health

## Abstract

**Background:**

Persisting symptoms after concussion (PSaC) are a pathological manifestation of head injuries that present with symptoms after the acute phase of head trauma has subsided. Insufficient research about PSaC has led to gaps in knowledge and incorrect terminology being applied. Furthermore, gaps exist in standardised assessment protocols and understanding of mental health symptoms associated with sports.

**Objectives:**

The study aimed to; 1) Determine expert consensus on appropriate terminology for symptoms lasting >4 weeks, 2) Investigate associations with mental health and postural stability symptoms, 3) Evaluate experts’ views on quantitative balance and electroencephalogram (EEG) testing.

**Methods:**

A Delphi-like survey was designed in REDCap and sent to identified experts in the field of sports-related concussions (SRC). Expert consensus was defined as ≥75% agreement.

**Results:**

Expert consensus identified the following mood and motor control symptoms being associated with PSaC: increases in emotional state (80%), irritability (87%), nervousness (87%), sadness (80%), balance impairment (80%), dizziness (87%) and feeling slow (80%). Numbness and tingling were not considered longer-term effects (80%). Additionally, 93% of respondents acknowledged mental health symptoms as potential longer-term effects, with 80% agreeing on inadequate current management. Respondents indicated PSaC are only somewhat adequately managed (73%) or not managed well enough (27%). The use of EEG and quantitative balance testing remains open for debate. The survey response rate was 21%.

**Conclusion:**

Improving mental health management for athletes with PSaC and standardising terminology is crucial. Future research is required to establish effective diagnosis and treatment methods. Addressing these issues may result in better care and safer return to play for athletes.

Persisting symptoms after concussion are a pathological manifestation of sports-related head injuries that present with signs and symptoms after the acute clinical symptoms of head trauma have subsided. ^[1–4]^ It has been suggested that persisting symptoms present in about 10%–30% of people depending on the specific population cohort and the time frames used to define prolonged symptom presentation. ^[5–7]^

Several attempts have been made to define persisting symptoms after concussion; however, a lack of consensus remains for a universally agreed upon terminology. ^[8]^ While it is sometimes referred to as post-concussion syndrome (PCS), it has been argued that this term should not be used, since ‘syndrome’ suggests that there is significant consistency in symptom presentation among those that are diagnosed. ^[1,8]^

A recent shift in terminology has begun to favour two terms, one being persistent post-concussion symptoms (PPCS), which describes a set of symptoms that presents after the clinical symptoms of a sports-related concussion (SRC) should have resolved. ^[8,5]^ The second being ‘persisting symptoms’, which describes a set of symptoms that present >4 weeks in children, adolescents and adults; this was recently termed in the 6th Consensus Statement on Concussion in Sport, held in Amsterdam in October 2022. ^[3]^ In some cases researchers have described PPCS as symptoms that can persist >3 months and have broken it up into differential phases (i.e. Phase 1: early phase post trauma presenting days to weeks after the injury has occurred; Phase 2: late phase post trauma presenting months to years post incident). ^[2,9]^

These persisting symptoms can generally be categorised into physical (i.e. postural instability, headaches, sleep disturbances, fatigue, neck pain), cognitive (i.e. attention deficit symptoms and impairment of executive functions) and emotional symptoms (i.e. irritability and symptoms of depression and anxiety); however, these are still inconclusive.^[10]^

Terminologies are still interchanged with one another in the clinical and research environment, creating confusion in discussions, literature and clinical settings. ^[2,8]^ It may lead to large variations in the diagnosis and treatment, placing the patient in a precarious situation with no clear return to sport criteria. In addition, the complexity surrounding testing and treatment of persisting symptoms poses a challenge for both the athlete and clinician, with the current gold standard of assessments still relying on standardised symptom rating scales. ^[5,8,11]^ A call for more accurate and replicable prognostic factors related to persisting symptoms after concussion is called for. ^[12]^ This may be due to the lack of evidence available to clinicians to assess and treat athletes presenting with such symptoms. ^[2,5,12]^ Currently, advances in neuroimaging and brain mapping techniques and utilising quantitative electroencephalogram (qEEG) are gaining traction in identifying regions of irregular brainwave activity in patients with persisting symptoms after concussion. ^[13]^

The management of the symptoms is considered interdisciplinary in nature. ^[5]^ Multiple practitioners with a clear understanding of their roles and responsibilities work with each other to manage the symptoms, and this is considered the current gold standard. ^[5]^ However, a definitive best practice management process for persisting symptoms is still lacking. By developing a universally conclusive definition and advocating for the correct usage of terminology to describe these persisting symptoms after concussion through identifying signs, symptoms, testing and treatment protocols, clinicians and researchers can aim to create clarity surrounding the complex topic.

The initial study’s objectives were to establish a more universally agreed upon terminology of the longer-term effects of SRC, what mental health and motor control signs and symptoms are suspected to be associated with persisting symptoms and if these are being addressed adequately. Also, whether electroencephalogram (EEG) and/or balance testing can be used as a diagnostic support tool for persisting symptoms, whether overall management of persisting symptoms is adequate, and in what direction future research should focus. The responses will help in developing future surveys surrounding this complex topic.

## Methods

### Study design

A mixed research design incorporating a cross-sectional Delphi-like survey was sent out to identified experts in the field of SRC with the use of REDCap (Research Electronic Data Capture).

### Participants

Experts were defined as individuals that displayed elevated levels of knowledge and experience (work-related experience or study expertise) in the given study and were determined by the following criteria: Is the individual currently involved with SRC research; Has the individual contributed academically to SRC; Does the individual teach on topics regarding SRC; Does the individual have formal training in concussion testing; Does the individual treat athletes that have suffered with SRC and persisting symptoms. All expert responses were ranked equally. The experts were identified via publications analysis, World Rugby’s list of approved independent concussion consultants and referrals from experts that completed the study. Prospective participants were emailed an information letter and a personalised link to the online survey. These links could not be forwarded to other potential participants; rather, contact had to be made with the researchers if a potential participant was referred.

### Ethical clearance

The research was approved by the Health Research and Ethics Committee (HREC) on the 1^st^ of March 2023. Reference number: S22/08/145 (PhD).

### Procedure

Data were collected and managed using REDCap electronic data capture tools hosted at Stellenbosch University. REDCap is a secure, web-based software platform designed to support data capture for research studies, providing; 1) an intuitive interface for validated data capture; 2) audit trails for tracking data manipulation and export procedures; 3) automated export procedures for seamless data downloads to common statistical packages; and 4) procedures for data integration and interoperability with external sources. Survey invitations were sent out as potential experts were identified and referred.

The Delphi-like survey consisted of 22 questions, which included Likert scales, lists, selections and open-ended questions exploring demographics, definitions, terminologies, diagnostics, signs, symptoms, treatment and management of SRC and persisting symptoms. The survey was designed by consulting and reviewing previous literature on the above topics (supplementary file).

### Statistical analysis

For our primary objective, descriptive statistics (average, Descriptive statistics and measures of central tendency, including means and standard deviations and frequencies, are provided for quantitative data. Normalcy of distribution was checked wherever applicable. In addition, qualitative open-ended responses from the experts were synthesised, summarised and discussed. Quantitative results were analysed in Microsoft^®^ Excel^®^ using the built-in data analysis plug-in tool. A consensus on questions asked was defined by ≥75% of experts agreeing.

## Results

### Demographics

The participants comprised local and internationally identified experts in the field of sport-related concussion. Eighty experts were identified and emailed an invitation to participate in the survey. Ten emails (13%) returned, citing “failure to deliver”. Of the 70 invitations (88%) that were successfully delivered, 15 responded (21% response rate). The overwhelming majority of respondents provided additional comments at every option where it was offered, with a mean optional response rate of 11.8 ± 1.4 additional comments. Of the 15 respondents (100%), two (13%) identified as primarily researchers, nine (60%) as sports physicians, one (6.7%) as a neurologist and three (20%) as both a sports physician and researcher. The general geographical distribution of the respondents included South Africa (40%), Wales (6.7%), the USA (40%) and the United Kingdom (13%).

The respondents’ mean experience in the field was 22.5 ± 11.6 years. The most experienced expert had worked in the field for 41 years, while the least experienced expert had worked in the field for three years.

### Persisting symptoms terminology

All respondents indicated that they had encountered the term “Persistent Post-concussion Symptoms (PPCS)”. When asked to choose which of the current definitions and terminology best described the longer-term effects of concussion, ten (67%) respondents selected the 6^th^ International Consensus Conference on Concussion in Sport, and five (33%) respondents selected “none of the above”. Persistent post-concussion symptoms can be defined as a set of symptoms that presents after the clinical symptoms of a SRC have resolved; this is universally agreed upon as being symptoms that persist >4 weeks. ^[1,3]^

### Usefulness of electroencephalogram and quantitative balance testing in athletes with persisting post-concussion symptoms

Only one respondent (6.7%) indicated that the EEG testing might be useful when screening athletes presenting with persisting symptoms, suggesting that modalities such as functional MRIs and EEG recordings might be able to detect slight disturbances in brainwave function that may predict the development of these symptoms. Six (40%) respondents were unsure of EEG usage in the population, citing that it may only be applicable in selected cases (e.g. if a specific neurological condition is suspected) and that only certain electrophysiological indicators may be useful; if it is utilised as an adjunct diagnostic tool or if it is utilised to demonstrate group-level differences in brainwave activity. Eight (53%) respondents said that EEG would not be useful.

Eight (53%) respondents saw benefit in balance testing for athletes with persisting symptoms, naming Balance Error Scoring System (BESS) and Modified Balance Error Scoring (mBESS) as strong markers for recovery from concussion and commenting that the added use of accelerometers may guide clinicians towards identifying more subtle abnormalities and discrepancies in motor control. The cost and access to specialised equipment posed a challenge to a wider use. Three (20%) respondents did not see the benefit in balance testing (especially beyond the sub-acute phase) since balance impairment typically resolves during the acute period of a concussion, and persistent impairment is generally subtle and of limited clinical value. Four (27%) respondents were unsure of the use of balance testing.

**Fig. 1:**
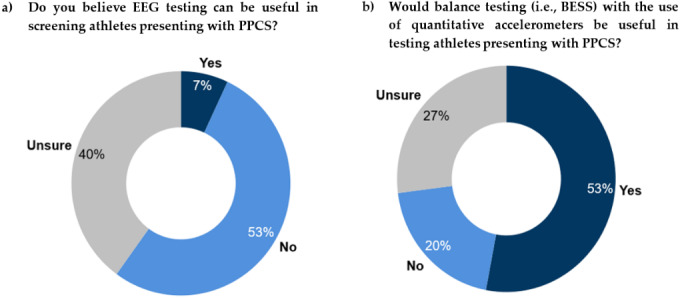
Perceived usefulness of EEG (a) and quantitative balance (b) testing in athletes with persisting post-concussion symptoms. EEG, electroencephalogram; PPCS, persisting post-concussion symptoms; BESS, balance error scoring system.

Figure 1 presents the responses regarding the perceived value of EEG and quantitative balance testing.

### Mood and motor control symptoms associated with the longer-term effects of concussion

**Fig. 2:**
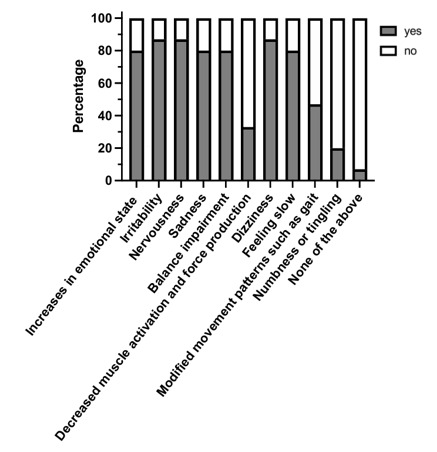
Mood and motor control symptoms associated with the long-term effects of concussion.

A consensus (>75%) was reached on the following mood and motor control symptoms believed to be longer term effects of concussion: increases in emotional state (80%), irritability (87%), nervousness (87%), sadness (80%), balance impairment (80%), dizziness (87%) and feeling slow (80%). Furthermore, a consensus was reached that numbness and tingling were not a longer-term effect of sport-related concussion (80%). A consensus was not reached on the following mood and motor control symptoms: decreased muscle activation and force production with five (33%) of the respondents saying yes and ten (67%) of respondents saying no; modified movement patterns such as gait with seven (47%) of respondents saying yes and eight (53%) of respondents saying no (Figure 2).

### Mental health symptoms

Fourteen (93%) respondents believed that depression and anxiety can be longer-term effects of SRC. When prompted to rate how well they believed that mental health symptoms such as depression and anxiety were managed in athletes with persisting symptoms, three (20%) respondents stated it is completely overlooked, twelve (80%) respondents selected “Managed however, improvement is needed”, and none believed these symptoms were managed sufficiently well. All respondents agreed that mental health symptoms have an impact on an athlete’s ability to perform optimally in a physically demanding environment. Furthermore, ten (67%) respondents agreed that athletes who are experiencing mental health symptoms post-concussion have a higher risk of suffering other sports-related injuries. Two (13%) respondents said no, while another three (20%) respondents were unsure or did not provide an answer.

Eleven (73%) of the respondents suggested that depression and anxiety in persisting symptoms are only “somewhat” adequately managed and treated in athletes; a further four (27%) respondents suggested that it is not managed adequately at all.

## Discussion

The current study set out to establish an expert consensus on topics related to the longer-term effects of concussion. It focused on choosing relevant current terminology, the association of mental health and postural stability symptoms within it, and the use of EEG and quantitative balance testing for it. To our knowledge, this is the first expert survey on the topic.

### Establishing uniformity in terminology

Various terminology for longer-term presentations and symptoms of concussion is still interchangeably used in the research and clinical setting, including post-concussion syndrome, along with persistent or persisting post-concussion or -concussive symptoms, although the latest Consensus Statement on concussion in sports postulates the most current definition and terminology to use.^[3, 2,10,8,9]^ The need for raising awareness of the correct terminology has become especially evident in the current survey where the researchers synonymously used the words ‘persistent’ and ‘persisting’ and defined the longer-term effects of the concussion as PPCS. While a consensus was not reached in this study, 67% of the respondents believed that the latest definition and terminology of persisting symptoms after concussion provided by the 6th Consensus Statement was the most appropriate in describing the longer-term presentations after suffering a sports-related concussion. ^[3]^ Importantly, two of the respondents emphasized the importance of using the term “persisting symptoms” as it reflects a major emphasis that the symptoms are treatable even though they are present beyond the typical time period. ^[1]^

### Decoding the clinical framework of persisting symptoms

**Fig. 3:**
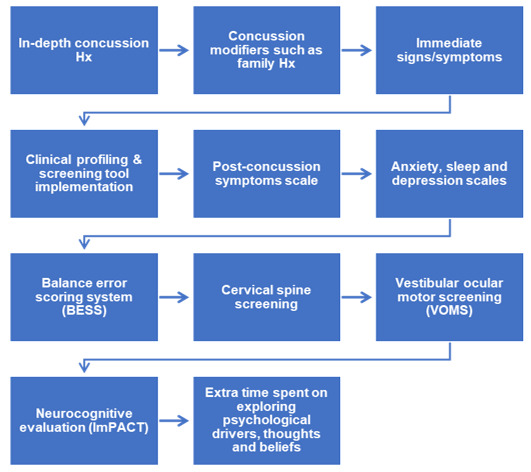
Suggested general pipeline for concussion evaluation. Hx, history; ImPACT, immediate post-concussion assessment and cognitive testing.

Detailed knowledge on SRC pathophysiology is currently limited to the acute phase with gaps presenting for persisting symptoms (i.e. symptoms lasting >4 weeks). ^[14]^ This study has explored what testing procedures can be implemented in persisting symptoms to identify linked biomarkers that have replicability in a clinical setting. ^[3]^ A general concussion evaluation pipeline suggested by one of the respondents (Figure 3) provides a foundation for the investigation and future clinical implementation.

### Brainwave activity

Concussion research has indicated that the most common areas of the brain associated with SRCs include the anterior regions (i.e. frontal lobe and anterior cingulate) which display reduced activity after injury as well as the cerebellum and parietal lobe, which display increased neural activity and foci. ^[15]^ With that in mind, identifying brainwave activity biomarkers has shown promise as a diagnostic tool for SRCs and persisting symptoms most noticeably when the raw electrical brainwave activity is combined with mathematical software-assisted analysis in quantitative electroencephalogram (qEEG) testing. ^[15]^ Uncertainty persists with brainwave activity monitoring with the use of EEG as a biomarker for SRCs and persisting symptoms, this was evident with 40% of the respondents voicing uncertainty with regards to its usage in a clinical setting (Figure 1). This may be a result of knowledge gaps and experience using the diagnostic tool and the experimental nature that quantitative neuroimaging research finds itself in with regard to the topic at hand. ^[3]^

### Postural stability

Postural control deficits, specifically postural instability, are commonly observed in persisting symptoms after concussion.^[16,17]^ Numerous studies suggest using subjective balance screening tools as part of the diagnostic toolbox (i.e. Sport Concussion Assessment Tool 6 (SCAT-6^TM^) and Sport Concussion Office Assessment Tool 6 (SCOAT-6^TM^) assessment toolboxes). ^[3,16–18]^ This is due to findings that suggest SRC can cause dysfunction in postural stability that presents well after the initial traumatic event. ^[16,17]^ Two of the most commonly utilised screening tools for postural discrepancies after concussions are the Balance Error Scoring (BESS) and the Modified Balance Error Scoring System (mBESS). ^[3,16–18]^ Both tests challenge the athlete to stand in certain balancing positions (double stance, single-leg stance and tandem stance) with their eyes closed on firm (mBESS) and/or foam surfaces (BESS) for 20 seconds, without making subjectively identified errors (i.e. hands lifted off iliac crest; opening eyes; step, stumble, or fall; moving hip into >30° abduction; lifting forefoot or heel; remaining out of test position >5 seconds). ^[3]^

These tests are considered the current gold standard for testing postural instability in SRC; however, further room for accuracy and quantification may be required to better establish postural stability as a replicable biomarker in the sporting population. ^[16]^ This was emphasised with 53% of respondents suggesting that balance testing with quantitative accelerometers (e.g., APDM Mobility Lab^TM^ software and body-worn sensors^©^) may provide a more detailed insight into postural instability within the population. At the same time several respondents expressed uncertainty due to the possible costs involved in its implementation into a clinical setting and the lack of studies validating their use. While an overwhelming consensus was not achieved for using balance tests with an accelerometer, only 20% of respondents refuted its benefit (Figure 1). Interestingly, most respondents believed balance impairments to be a symptom of the longer-term effects of concussion; however, just over half of the experts believed balance testing with quantitative accelerometers could be useful in testing in the same population.

### Motor control

Changes in the brain, including electrical activity, metabolic balance, oxygen consumption and irregular cerebral blood flow, continue for a prolonged period after concussion, increasing the risk of repeat concussions, psychological disorders, or musculoskeletal injuries. ^[18,19]^ It has been found that athletes who had sustained an SRC and returned to play displayed a higher incidence rate of musculoskeletal injuries compared to athletes who had not. ^[20]^ In addition, suspected dysfunction of sensorimotor integration due to disrupted neural connections and axonal shearing may lead to motor control deficits (i.e. postural instability, reduced muscle activation and force output, dizziness, peripheral neurological symptoms, modified gait patterns, and feeling of being slow etc.) that might lead to worsened performance. ^[19]^

Symptom checklists such as the Symptom Evaluation Checklist found in the SCAT6/SCOAT-6 and the ImPACT PCSS form a base of testing for athletes with SRC and persisting symptoms. ^[3,21]^ Both evaluate similar areas of interest; however, neither of them explores in detail subjective motor control signs and symptom deficits such as gait impairment and reduced muscle activation and force production (i.e. feeling physically weak). A consensus was not reached on the former, suggesting further research may be required to determine if these variables should be subjectively screened for during athlete evaluation.

### Mental health

Mental health variables, including depressive mood and anxiety-like symptoms, have been associated with persisting symptoms; however, the findings remain inconclusive. ^[22]^ Psychological factors have also been identified as predictors for musculoskeletal injuries, therefore, raising the question of whether psychological factors and symptoms caused by concussions in sport may have a similar causation and have detrimental effects on the musculoskeletal system. ^[22]^ Ten (67%) of the respondents believed this to be the case.

A consensus was reached that mental health symptoms can be longer-term effects of SRC, even though caution should be taken when attempting to correlate these with SRC as these may be pre-existing before the injury and that the concussion itself may have “unmasked” and/or exacerbated the already present symptoms. ^[3]^

An overwhelming majority of respondents noted that persisting symptoms in general, and mental health in persisting symptoms particularly, are currently not sufficiently well managed. They provided the following recommendations for improvement:

- Early and established recognition and exercise rehabilitation
  ○ Ensure early physical activity/exercise rehabilitation
  ○ Minimise work or academic accommodations
  ○ Restore behavioural routine including regular sleep (no napping), hydration, nutrition, stress minimisation
  ○ Taking note of critical contributions of pre-existing, coexisting and resulting mental health issues in concussed athletes with persisting symptoms, avoiding attributing all persisting symptoms after a concussion solely to the concussion itself
  ○ Identify and treat co-existing vestibular issues, as these may increase anxiety like symptoms
  ○ Consider medication with combined psychological treatment if symptoms do not subside
- Education and counselling:
  ○ Baseline education for the athletes and coaching staff on the impact concussions may have on a player’s mental state, on the symptom checklists (SCOAT-6 provides anxiety, depression, sleep screening and fatigue tools) and on the importance of referring to mental health practitioners
  ○ Normalize discussions around the topic to reduce the associated stigma
  ○ Encourage maintenance of team and social interaction
  ○ Avoid creating an environment of fear and caution by withholding athletes from physical activity, increased time spent in testing and mismanagement of the athletes’ social interactions

### Other biomarkers: Avenues for future research

Since currently no biomarkers have been validated for the diagnostics of SRC and persisting symptoms, these areas for future research are suggested:

- Psychological symptoms and mood disorders as these have been determined to be a strong predictor for persisting symptoms after concussion
- Motor control exercise tolerance testing
- Vestibular-oculomotor tools and screening
- Diffusion-weighted magnetic resonance imaging (DW-MRI)
- Measurement and monitoring of the following proteins and enzymes: Glial fibrillary acidic protein (GFAP); ubiquitin C-terminal hydrolase-L1 (UCH-L1); calcium-binding protein S100, beta isoform (S100β); myelin basic protein; neuron-specific enolase (Gamma-enolase); and prostaglandin D synthase
- Salivary and messenger ribonucleic acid (mRNA) samples

### Limitations

Several limitations are present in the current study that are usually associated with Delphi-like surveys including; expert selection bias, the lack of standardisation on data interpretation and analysis, the reliance on the experts’ knowledge and willingness to contribute and share their opinions, the risk of the questions simplifying the complexity surrounding SRC and persisting symptoms, the lack of face-to-face interaction between the experts to discuss the questions at hand and the risk of group thinking taking place limiting the exploration of unique and novel thought processes.

In addition, a limitation of this study is the lack of multiple survey rounds to address questions and topics that have not reached the ≥75% consensus. The current Delphi-like survey also had numerous objectives, which may have created overcomplexity in the study, and future research should aim to narrow its focus. More clarity may have also been useful to minimise potential confusion in the questions exploring depression and anxiety as part of the symptom complex of persisting symptoms after concussion. Finally, this study forms only a small aspect of the complexities that present in persisting symptoms after concussion, exploring a select few signs and symptoms and potential testing and treatment modalities.

An attempt was made to address the above limitations by including experts from diverse geographical regions and backgrounds as well as structuring thought provoking questions that challenged the current narrative of SRC and persisting symptoms after concussion.

## Conclusion

While expert consensus on numerous topics surrounding persisting symptoms is still open, research is moving in the correct direction. ^[1,7,11,16,23]^ This study identified several areas where improvement is needed, from the identification of causes of mental health and motor control symptoms through early diagnosis and individualised management up to long-term patient follow-up. Furthermore, attention and training at an undergraduate level for health and medical practitioners to better identify signs and symptoms of persisting symptoms after concussion were called for.

At the same time, several positive steps have already been taken, including an increased focus on early implementation and activation of both physical and cognitive screenings and interventions through the use of tools such as the newly developed SCAT-6 and SCOAT-6.

Future studies should focus on randomised control trials (RCT) that explore the effects of interventions such as exercise and attempt to develop best practice guidelines, early identification and comprehensive treatment of psychosocial factors associated with SRC and persisting symptoms after concussion. ^[23]^ Athlete risk profiling needs to be addressed by implementing biomarkers such as postural abnormality discrepancy. The search for biomarkers that are correlated with SRC and persisting symptoms should continue, keeping in mind that, due to the complexity of brain injuries, it is unlikely that one marker will be relevant for all cases. ^[23]^ Finally, research should explore specific populations (i.e. age, gender etc.) as symptoms and recovery times may differ. ^[23]^

It is also recommended that Delphi and Delphi-like surveys limit their focus to a select few variables to avoid creating overcomplexity. In addition, pre-surveys can be sent out first to determine if a need exists for exploring the topic at hand and implementing follow-up strategies, such as phone calls, to improve the response rate.

## Data sharing statement

The study’s raw data and relevant supporting materials will be made available to other researchers upon request. Request for access to the data should be directed to the corresponding author. Raw data will be anonymised and password-protected before being shared.

## Supplementary Information



## References

[1] Broshek DK, Pardini JE, Herring SA (2022). Persisting symptoms after concussion: Time for a paradigm shift. PM&R.

[2] Jennings T, Islam MS (2023). Examining the interdisciplinary approach for treatment of persistent post-concussion symptoms in adults: a systematic review. Brain Impair.

[3] Patricios JS, Schneider KJ, Dvorak J, Ahmed OH, Blauwet C, Cantu RC (2023). Consensus statement on concussion in sport: the 6th International Conference on Concussion in Sport–Amsterdam, October 2022. Br J Sports Med.

[4] Silverberg ND, Iverson GL, Cogan A, Dams-O-Connor K, Delmonico R, Graf MJP (2023). The American Congress of Rehabilitation Medicine Diagnostic Criteria for Mild Traumatic Brain Injury. Arch of Phys Med Rehabil.

[5] Makdissi M, Schneider KJ, Feddermann-Demont N, Guskiewicz KM, Hinds S, Leddy JJ (2017). Approach to investigation and treatment of persistent symptoms following sport-related concussion: a systematic review. Br J Sports Med.

[6] Sandel N, Reynolds E, Cohen PE, Gillie BL, Kontos AP (2017). Anxiety and mood clinical profile following sport-related concussion: From risk factors to treatment. Sport Exerc Perform Psychol.

[7] Langdon S, Goedhart E, Inklaar M, Oosterlaan J, Königs M (2023). Heterogeneity of persisting symptoms after sport-related concussion (SRC): exploring symptom subtypes and patient subgroups. J Neurol.

[8] Dwyer B, Katz DI (2018). Postconcussion syndrome. Handb Clin Neurol.

[9] Katz DI, Cohen SI, Alexander MP (2015). Mild traumatic brain injury. Handb Clin Neurol.

[10] Rytter HM, Graff HJ, Henriksen HK, Aaen N, Hartvigsen J, Hoegh M (2021). Nonpharmacological Treatment of Persistent Postconcussion Symptoms in Adults: A Systematic Review and Meta-analysis and Guideline Recommendation. JAMA Netw Open.

[11] Yeates KO, Räisänen AM, Premji Z, Debert CT, Frémont P, Hinds S (2023). What tests and measures accurately diagnose persisting post-concussive symptoms in children, adolescents and adults following sport-related concussion? A systematic review. Br J Sports Med.

[12] Déry J, Ouellet B, De Guise É, Bussières ÈL, Lamontagne ME (2023). Prognostic factors for persistent symptoms in adults with mild traumatic brain injury: an overview of systematic reviews. Syst Rev.

[13] Munia TTK, Haider A, Schneider C, Romanick M, Fazel-Rezai R (2017). A Novel EEG Based Spectral Analysis of Persistent Brain Function Alteration in Athletes with Concussion History. Sci Rep.

[14] Manley G, Gardner AJ, Schneider KJ, Guskiewicz KM, Bailes J, Cantu RC (2017). A systematic review of potential long-term effects of sport-related concussion. Br J Sports Med.

[15] Ianof JN, Anghinah R (2017). Traumatic brain injury: An EEG point of view. Dement Neuropsychol.

[16] Kerr HA, Ledet EH, Hahn J, Hollowood-Jones K (2022). Quantitative Assessment of Balance for Accurate Prediction of Return to Sport From Sport-Related Concussion. Sports Health.

[17] Reilly N, Prebor J, Moxey J, Schussler E (2020). Chronic impairments of static postural stability associated with history of concussion. Exp Brain Res.

[18] Purkayastha S, Adair H, Woodruff A, Ryan LJ, Williams B, James E (2019). Balance Testing Following Concussion: Postural Sway versus Complexity Index. PM R.

[19] Kilic Ö, Hopley P, Kerkhoffs GMMJ, Lambert M, Verhagen E, Viljoen W (2019). Impact of concussion and severe musculoskeletal injuries on the onset of mental health symptoms in male professional rugby players: a 12-month study. BMJ Open Sport Exerc Med.

[20] McPherson AL, Nagai T, Webster KE, Hewett TE (2019). Musculoskeletal Injury Risk After Sport-Related Concussion: A Systematic Review and Meta-analysis. Am J Sports Med.

[21] Riegler KE, Guty ET, Arnett PA (2019). Validity of the ImPACT Post-Concussion Symptom Scale (PCSS) Affective Symptom Cluster as a Screener for Depression in Collegiate Athletes. Arch Clin Neuropsychol.

[22] Rice SM, Parker AG, Rosenbaum S, Bailey A, Mawren D, Purcell R (2018). Sport-Related Concussion and Mental Health Outcomes in Elite Athletes: A Systematic Review. Sports Med.

[23] Kamins J, Bigler E, Covassin T, Henry L, Kemp S, Leddy JJ (2017). What is the physiological time to recovery after concussion? A systematic review. Br J Sports Med.

