# Supplementary material for: Expert consensus on the terminology, diagnostics and management of persisting symptoms after concussion with a focus on mental health, postural stability, electroencephalogram and balance testing: A cross-sectional Delphi-like survey
Source: S Afr J Sports Med. 2024 Jul 15;36(1):v36i1a17870. doi: 10.17159/2078-516X/2024/v36i1a17870 (PMC11294671; doi:10.17159/2078-516X/2024/v36i1a17870)
Supplement: Supplementary file 1 [file 2078-516X-36-v36i1a17870-s001.pdf]

Expert Consensus Persistent Post-concussive Symptoms

We would like to invite you to take part in a research project which involves the completion of an online survey. Your participation is entirely voluntary, and you are free to decline to participate or to stop completing the questionnaire at any time, even if you have agreed to take part initially. The initial results of the questionnaire may be shared with other researchers, health professionals and sports managers in rugby who have completed the same survey as well as potential academic journals for future publications. Subsequent questions may be asked to develop further clarity on the topic.

The following survey will set out to explore the current state of knowledge and attitudes surrounding persistent post-concussive symptoms (PPCS), mental health symptoms and motor control. It will also set out to provide a more consistent and universally accepted definition of persistent post-concussive symptoms.

Please complete the survey below.

Kind Regards

Michael James Lumb

PhD Candidate (Sports Science), MPhil (Biokinetics) [ORCID-ID: 0000-0002-4605-2530]

If you consent to partake please sign where indicated. ______________________

Name and Surname (Optional): ______________________

Please share your email address if you do not mind being contacted by the researchers of this study in the future (Optional): ______________________

General Background Questions

1 In what role do you specifically work with sport-related concussions? ______________________

2 How many years have you been involved in the sporting environment? ______________________

Persistent Post-concussive Symptoms (PPCS)

3 Have you come across the term of PPCS?

- ◯ Yes
- ◯ No

4 Which of the definitions listed below do you believe best describes PPCS?

◯ 1 ◯ 2 ◯ 3 ◯ 4 ◯ 5

1) ICD-11 (International Statistical Classification of Diseases and Related Health Problems): ICD-11 does not use the term post-concussion syndrome and rather described the condition as mild neurocognitive disorder that presents < 1month between head injury and symptom onset. Symptoms include physical (headaches, dizziness, fatigue, insomnia, noise intolerance), emotional (irritability, reduced alcohol tolerance, depression, anxiety, emotional liability, preoccupation with symptom), and cognitive (decline in concentration, memory, or intellectual difficulties) presentations.

2) (DSM)-V (Diagnostic and Statistical Manual of Mental Disorders fifth edition): The fifth addition does not use the term post-concussion syndrome and rather describes the condition as "major or mild neurocognitive disorder due to traumatic brain injury". The definition requires evidence of impact to the head or other mechanical trauma resulting in impact to the brain as well as one or more clinical findings including: loss of consciousness, amnesia post injury, disorientation and concussion, clinically or neurological imaging detection of new onset or worsening of symptoms including seizures, visual disruptions, anosmia and/or hemiparesis. The disorder should develop immediately after injury or after consciousness has been regained and should persist past the acute phase of the injury.

3) 6th International Consensus Conference on Concussion in Sport: Persistent symptoms should only be used when an individual (including children, adolescents and adults) present with symptoms for >4 weeks post injury. These symptoms are persistent symptoms experienced during the acute stage of a sport relate concussion including: loss of consciousness, amnesia, neurological deficits, balance impairments, behavioural changes and cognitive deficits.

4) None of the above, a consolidated, universal and clearer definition is required, containing for example specific symptom criteria, cut-off criteria, duration of symptom presentation and specific biomarkers (i.e., EEG brainwave abnormalities).

5) None of the above.

---

5 Do you believe electroencephalogram (EEG) testing can be useful in screening athletes presenting with PPCS?

◯ 1) Yes
◯ 2) No
◯ 3) Unsure

5.1 Any additional comments on the above EEG question can be made here.

____

6 Would balance testing (i.e., The Balance Error Scoring System (BESS)) with the use of quantitative accelerometers be a useful in testing athletes presenting with PPCS?

- ◯ 1 Yes
- ◯ 2 No
- ◯ 3 Unsure

6.1 Any additional comments on the above balance testing question can be made here.

____

7 What other biomarkers do you believe should be screened for in athletes with PPCS?

____

Mental Health Symptoms and Motor Control in Persistent Post-concussive Symptoms.

8 Which of the following mood and motor control symptoms do you believe can be longer term effects of concussions?

- ☐ Increases in emotional state
- ☐ Irritability
- ☐ Nervousness
- ☐ Sadness
- ☐ Balance impairment
- ☐ Decreased muscle activation and force production
- ☐ Dizziness
- ☐ Feeling slow
- ☐ Modified movement patterns such as gait
- ☐ Numbness or tingling
- ☐ None of the above

8.1 What other mood and motor control symptoms do you consider longer term effects of concussion that were not mentioned above?

____

9 Do you believe that mental health symptoms such as depression and anxiety can be a longer term effect of concussions?

- ◯ Yes
- ◯ No
- ◯ Unsure

10 How well do you believe that mental health symptoms such as depression and anxiety are managed for athletes with PPCS?

Completely overlooked — Managed however improvement needed — Managed sufficiently well

(Place a mark on the scale above)

11 To your knowledge, how are mental health symptoms currently managed in athletes?

____

12 How would you suggest the mental health symptoms be managed (for example, specific medical treatment or professional intervention)?

____

13 Do you believe that mental health symptoms have an impact on an athlete's ability to perform optimally in a physically demanding environment?

- ◯ Yes
- ◯ No
- ◯ Unsure

13.1 Any additional comments on the above question can be made here.

____

14 Do you believe that an athlete who is experiencing mental health symptoms post-concussion is at a higher risk of suffering other injuries (i.e., orthopaedic injuries)?

◯ Yes
◯ No
◯ Unsure

14.1 Any additional comments on the above question can be made here.

____

The Management of Persistent Post-concussive Symptoms and Future Research

15 Overall do you believe that PPCS is adequately managed and treated in athletes?

Not at all   Somewhat   Thoroughly

(Place a mark on the scale above)

16 Based on your answer above what do you believe is being implemented well and what can be improved on?

____

17 In what direction do you believe PPCS research should be focused on and why?

____
